# Prevalence and Management of Metabolic Syndrome in Adult Psychiatric Patients Receiving Second-Generation Antipsychotics at Sheikh Khalifa Medical City

**DOI:** 10.7759/cureus.22021

**Published:** 2022-02-08

**Authors:** Wadha Mohammed, Waeil Al Naeem

**Affiliations:** 1 Pharmacy, Sheikh Khalifa Medical City, Abu Dhabi, ARE

**Keywords:** sheikh khalifa medical city, second-generation antipsychotics, psychiatric patients, adult, management of metabolic syndrome

## Abstract

Introduction

The establishment of second-generation antipsychotics in the early 1900s has shown greater benefits in many outcome domains. As atypical antipsychotics revealed an improvement in the shortcomings of first-generation antipsychotics, they also have significant limitations in terms of side effects.

Background

As a class, atypical antipsychotics have a more satisfactory profile in terms of extrapyramidal side effects but have other side effects, including metabolic syndrome. Moreover, a patient with severe mental illness has reduced life expectancy compared to the general population and increased dying risk by two to three folds due to the associated risk of metabolic syndrome. Metabolic syndrome induced by second-generation antipsychotics requires routine monitoring, suitable intervention, and management, including lifestyle modification, caregiver education, and the use of other medications like lipid-lowering agents, anti-diabetics, and adjunctive therapies.

Method

This is a retrospective observational study that involves a review of medical records of adult patients attending the psychiatry outpatient clinics in Shaikh Khalifa Medical City to whom oral second-generation atypical antipsychotics were prescribed between January 2016 and December 2017. All pediatric patients were excluded, as well as those who are on other agents known to induce metabolic syndrome, including first-generation antipsychotics, mood stabilizers, anxiolytics, and anti-depressants. Besides, a patient who already developed metabolic syndrome before starting atypical antipsychotics was excluded as well. IBM SPSS v. 25 (IBM Corp., Armonk, NY) and Jamovi 1.0.4.0 (The jamovi project (2021). [Computer Software]. Retrieved from https://www.jamovi.org) were used for statistical analysis. The Shapiro-Wilk was used to test for normality of data, One-way repeated measure analysis of variance (ANOVA) was used to determine whether there are any statistically significant differences between the means of BMI across the three pre-specified time points, Kendall’s tau was used to test the correlation between categorical variables, and the paired t-test was used to determine whether there was a statistically significant mean difference between BMI at baseline and after one year, a p-value above 0.05 is considered non-significant.

Result

A total of 123 patients were included in this study out of 4123 patients. An olanzapine disintegrating tablet was the most used atypical antipsychotic among the study population, followed by risperidone and quetiapine, respectively. Furthermore, BMI was statistically significantly increased from baseline to six months (M = 2.37 kg/m^2^, p = .002), and from baseline to 12 months (M =3.26 kg/m^2^, p < .001) but not from six months to 12 months (M =1.41 kg/m^2^, p = .346). Due to lack of documentation and monitoring, it was difficult to assess the continuous change in total cholesterol level/high-density lipoprotein, glucose, and HbA1C level. It was observed that nine (7.32%) patients of those who have been included in the study started a treatment to manage the metabolic syndrome.

Conclusion

Patients on second-generation antipsychotics must be monitored and treated in case of metabolic syndrome development to decrease the high risk of mortality in such patients. Documentation of such complications, patient treatment, and progress is essential for outcome improvement.

## Introduction

Psychology and mental health services in the United Arab Emirates

The population of the United Arab Emirates (UAE) is estimated to be 9.4 million people, with an increase of 2.71 percent annually [1]. UAE is ranked the 21st in world population growth. Nevertheless, with the population growth, there are suspicions that mental health services and the general field of psychology have not matched the country's expansion, with a resulting surge in the level of unmet needs. The federal government's Ministry of Health (MOH) is the authorized body that manages health care legislation in the UAE. However, two other semi-governmental bodies, the Abu Dhabi Department of Health (DOH) and Dubai Health Authority (DHA) regulate their respective emirates. Currently, an independent mental health policy does not exist, but a mental health plan is included in the general health policy, according to the World Health Organization (WHO). The field of Psychology in the UAE began in the 1970s with the establishment of an undergraduate Psychology program in the United Arab Emirates University. A panel of professionals from DOH, DHA, MOH, Emirates Psychological Association, and other institutes are working on regulation guidelines for the practice of Psychology. Although there is an ongoing effort to establish a regulatory body for the professions of psychology in the UAE, there are still many challenges hindering the progress especially with licensing procedures and licensing sources (federal versus local) [2].

The mental health plan was reviewed in 2010 with an emphasis on the broader availability of mental health services in government facilities across the country. There is a shift toward incorporating mental health services into primary care and allocating more resources to a smaller community of mental health facilities. Data from governmental mental health facilities in 2011 specify that there were three outpatient facilities and mental health hospitals. There were also 25 beds for psychiatric patients in the general hospitals, and 80 beds available in the specialized mental hospital. The rate of mental health professionals per 100,000 was as the following: 0.3 psychiatrists, 0.51 psychologists, 0.25 social workers, 0.04 occupational therapists, and 0.04 other health workers. These numbers indicated an extreme shortage of mental health professionals and facilities in the UAE. In 2014, reports indicated that there were approximately 33,000 mental illness patients in the country and new admissions to public facilities on waiting lists that can reach up to two months [2].

The Emirates Psychological Association (EPA) was established in 2003 in Dubai by the Ministry of Social Affairs. It is the only officially recognized professional association for psychologists in the UAE. The EPA’s mission is to raise awareness of mental health issues in the Emirates and to advocate for the public’s access to mental health services. Besides that, it is serving as a liaison between governmental departments and private sectors to ensure better service to the community. Training psychologists in UAE is very challenging. Most of the Emiratis who provide psychological services in schools and the community mental health agencies have received minimal training in applied psychology. The only well-established public undergraduate program is available in the United Arab Emirates University (UAEU), which admits a very limited number of international students. This is because UAEU is a federal institution funded by the government. Also, Zayed University (ZU), another federal institution, recently began offering a psychology program as a major.

Expat students seeking training in Psychology have to enroll in private universities, such as Middlesex University Dubai and Heriot-Watt University Dubai, which also offers undergraduate programs in Psychology. Other universities, such as New York University Abu Dhabi and American University Sharjah, offer minors in Psychology or hybrid majors involving Psychology. At the graduate level, only one clinical Psychology Masters (MSc) program exists, which was recently introduced in UAEU, and it is open for both UAE nationals and non-nationals [2].

Mental health is a considerably neglected area of research in the UAE and the lack of attention to mental health research is a regional phenomenon affecting all of the Gulf Cooperation Council (GCC) countries. Through the period between 1989 and 2008, only 192 studies on mental health illness were published in the GCC countries. Among these countries, the UAE is the most prolific. The available studies indicated a high prevalence of psychosomatic disorders, depressive disorders, and anxiety disorders among primary health care attendees. Between the local geriatric population, the dominant mental disorders were depression, anxiety, and hypochondriasis. The authorities have to release and enforce the application of guidelines regulating the practice of Psychology, to protect the integrity of the profession and protect patients [2].

Behavioral Sciences Pavilion

Psychiatric care and services are provided in the Behavioral Sciences Pavilion (BSP). The BSP is a multidisciplinary mental health provider within SKMC. One of these disciplines is the Psychology department, which offers various psychological services, both inpatient and outpatient. BSP is serving 123 beds in both the male and female wards, including acute wards, long-stay wards, progress wards, chemical dependency units, and forensic units. While outpatient clinics are serving approximately 175 patients daily, they also offer services to a vast range of ages. The psychologists in the Child & Adolescence clinic offer testing and psychotherapy services. Patients are referred from various health providers, both within and outside of SKMC. The clinic also accepts referrals from schools and other non-healthcare organizations. While the General Psychiatry clinic offers the same services to ages from 18 to 65 years old, services to clients older than 65 are offered through the Geriatric clinic.

Moreover, BSP offers services in the Day Center and through the Case Community Management Team as well as Psychology services. Psychiatrists in BSP are dealing with the prevention, diagnosis, treatment, and rehabilitation of patients with mental and behavioral disorders. In order to achieve this role, psychiatrists possess a defined body of medical, and in particular psychopathological, knowledge and a defined set of procedural skills that are used to collect and interpret data, make appropriate clinical decisions, and carry out diagnostic and therapeutic procedures using an appropriate combination of biological, psychological, and sociological methods.

Mental illness treatment and complications

A patient with severe mental illness has reduced life expectancy compared to the general population. Also, the mortality risk is increased by two to three folds in such a population due to the associated risk of metabolic syndrome [3]. Recently, atypical antipsychotics used to treat such mental illness improved the outcomes and reduced neurological side effects compared to older antipsychotics. Hence, it became evident that atypical antipsychotic agents have a negative impact on some of the modifiable risk factors; part of these negative impacts can be clarified by the liability of some antipsychotics to induce significant weight gain, increased appetite, hyperglycemia, and excessive sleepiness [4]. A recent study of metabolic syndrome in patients diagnosed with schizophrenia between 2000 and 2006 as compared to 1984-1995 revealed that patients who received atypical antipsychotics had over twice the rate of new incident cases of metabolic syndrome after three years of treatment compared to those who were treated with conventional antipsychotic agents [5].

Moreover, clozapine and olanzapine, in particular, were the atypical antipsychotics carrying the highest risk of weight gain. However, newer agents of atypical antipsychotics, such as risperidone and aripiprazole, were considered to be less susceptible to cause weight gain [6]. Patients on second-generation atypical antipsychotics must be monitored for the associated metabolic side effects and other side effects, including weight gain, hyperlipidemia, diabetes mellitus, QT prolongation, and sexual side effect. Accordingly, the baseline of lipid profile, body mass index, fasting, and random glucose level should be monitored within the atypical antipsychotics use [7].

Home care services for a patient with mental illness

Home care services have been considered nowadays for a patient with long-term or acute mental illness who fulfills the criteria. The criteria necessitate that patients be stable, not living alone, and not tend to harm themselves and others. Additionally, the team that provides such services to patients with mental and behavioral disorders should be well trained and qualified. Professional training and qualification allow the nurses and the team to evaluate patient symptoms, monitor adverse effects and events, and educate the caregivers [8].

Schizophrenia: overview, epidemiology, and treatment

Schizophrenia is a chronic and severe mental disorder characterized by distortions in thinking, perception, emotions, language, sense of self, and behaviors. Patients diagnosed with psychosis are commonly experiencing hallucinations, delusions, and false beliefs. Worldwide, schizophrenia is associated with considerable disability and may affect educational and occupational performance. Additionally, schizophrenia has been correlated with an increased risk of diabetes since the nineteenth century. Maudsley was one of the first psychiatrists who noticed the association between diabetes and schizophrenia, and it was before the development of antipsychotics. Various studies have revealed that overweight and diabetes are, in general, increased two- to four-folds in schizophrenia patients as compared with the general population [9]. According to the World Health Organization (WHO), schizophrenia is affecting more than 21 million people worldwide. Out of those 21 million, schizophrenia is more common among males (12 million) as compared to females (9 million). Fifty percent (50%) of those patients are not receiving appropriate care while 90% of untreated patients are living in low to middle-income countries. Schizophrenia is usually managed by medications. However, management programs in low- and middle-income countries like Iran, Tanzania, Pakistan, India, and Ethiopia have demonstrated the likelihood of providing care to people with severe mental illness through the primary healthcare system by supporting families in providing home care, access to antipsychotics, public education, enhancing independent living skills, and training primary health care personnel.

Moreover, people with schizophrenia are less likely to seek care than the general population [10]. Schizophrenia is a highly heritable condition, which is associated with a significant reduction in lifespan, as one of the meta-analyses illustrated, and a substantial increase in mortality rate in psychosis patients compared to the general population. Hence, the mortality rate has worsened in the recent period due to the potential use of second-generation antipsychotic medications [11].

About 60% of patients with schizophrenia meet the criteria of metabolic syndrome in the United States of America compared to the general population who are at 30% risk of developing metabolic syndrome [12]. This association between metabolic syndrome and schizophrenia has been raised due to the use of antipsychotics. Antipsychotic drugs have been the backbone of schizophrenia treatment for approximately 50 years. Atypical antipsychotics are used to treat bipolar disorder and they have shown to be efficacious in treating both bipolar depression and manic phases [13]. Likewise, recent evidence points to the efficacy of second-generation antipsychotics for the treatment of bipolar depression [13]. Nevertheless, many patients diagnosed with psychosis receiving second or first-generation antipsychotics have had a suboptimal outcome, with symptomatic relapses and disabling adverse effects, predominantly, sedation and extrapyramidal symptoms. First-generation antipsychotics, or in other terms typical antipsychotics, act on the dopaminergic system by blocking dopamine type 2 receptors. However, this mechanism leads to a range of extrapyramidal side effects. While these antipsychotics are effective against the positive symptoms of schizophrenia, they have been considered to be ineffective in treating negative symptoms. Such symptoms predominantly play a critical role in producing the severe social disabilities experienced by a patient diagnosed with psychosis. The concern of finding antipsychotics to manage both the positive and negative symptoms of schizophrenia led to the reestablishment of second-generation antipsychotics in the early 1990s. When compared to first-generation antipsychotics, second-generation antipsychotics have shown more enormous benefits in many domains. Although second-generation antipsychotics were developed to improve the shortcomings of first-generation antipsychotics, they also have significant limitations in terms of side effects. As a class, they have a more satisfactory profile in terms of extrapyramidal side effects, but they produce other side effects, including dyslipidemia, diabetes, weight gain, and sexual dysfunction, which can constitute the components of metabolic syndrome [14]. Two systematic reviews showed that both first- and second-generation antipsychotics are generally equivalent in terms of efficacy against positive symptoms. Another study has shown evidence of the superiority of second-generation antipsychotics in terms of the treatment of negative symptoms, cognitive enhancement, fewer extrapyramidal side effects, as well as improved subjective experience and tolerability. Such therapeutic improvement has led to a general shift away from first-generation antipsychotics in the treatment of schizophrenia [15]. Finally, atypical antipsychotics represent an advancement in treating psychosis, chiefly because of their lower liability to cause extrapyramidal side effects.

First-generation antipsychotics vs. second-generation antipsychotics

Therapeutic differences between second and first-generation antipsychotics are not evident. A study published in the Canadian Journal of Psychiatry revealed that second-generation antipsychotics are favorable in terms of optimizing medication adherence, behavior, and quality of life [16]. On the other side, some second-generation antipsychotics (clozapine, amisulpride, risperidone, and olanzapine) are more effective than first-generation antipsychotics while others are not proven to be superior to first-generation antipsychotics. Likewise, some second-generation antipsychotics produce a better functional recovery than first-generation antipsychotics and are cost-effective for the reduction of hospitalization costs. Some researchers suggest that the property of blocking serotonin receptors, characteristic of most second-generation antipsychotics, accounts for superior efficacy. Many second-generation antipsychotics (ziprasidone, quetiapine, sertindole) seem to have about the same efficacy as first-generation antipsychotics, notwithstanding being potent serotonin receptor blockers. Focusing on amisulpride, although it is not a serotonin receptor blocker, it is more efficacious than first-generation antipsychotics. Furthermore, one of the meta-analyses on the raw data of the registrational studies of olanzapine and risperidone revealed that both medications were slightly superior to first-generation antipsychotics on positive symptoms but moderately superior on negative symptoms, cognitive symptoms, mood, impulse control, and improvement of many symptoms that were untouched by first-generation antipsychotics [17].

Comparison between second-generation antipsychotics

A randomized clinical trial indicates that weight gain liability varies across the different second-generation antipsychotics. Besides, clozapine and olanzapine treatment are associated with a significant risk of weight gain. On the other side, risperidone, quetiapine, amisulpride, and zotepine generally showed low to moderate-level clinically significant weight gain. Moreover, ziprasidone and aripiprazole are generally associated with minimal mean weight gain and the lowest risk of more significant increases. Focusing on lipid profile and glucose level changes, many published studies, including observational studies, large retrospective database analysis, and controlled experimental studies, including randomized clinical trials, noted that different second-generation antipsychotics are associated with different effects on glucose and lipid metabolism. Such studies revealed consistent evidence that clozapine and olanzapine treatment is associated with an increased risk of diabetes mellitus and dyslipidemia. However, pending further testing from preclinical and clinical studies, limited controlled studies support the hypothesis that clozapine and olanzapine may have a direct effect on glucose regulation independent of adiposity. Overall, the rank order of risk detected of the second-generation antipsychotics suggests that the differing weight gain liability of atypical agents contributes to the differing relative risk of insulin resistance, dyslipidemia, and hyperglycemia [18].

Home care services for psychiatric patients

Cognitive and functional inabilities in a patient cause behavioral problems and affect the level of burden of the caregiver. In order to reduce the burden of mental illnesses on individuals and their families along with treatment, psychosocial support and home care services are offered. The home care services must be in contact with the patients and their families to provide them with problem-solving skills and increase their level of tolerance. Home care services provide sustainability to health services and aim to develop, sustain, protect, and rehabilitate the patient. Home care also aims to enable people to remain at home rather than use residential, long-term, or institution-based nursing homes. Moreover, home care contributors deliver services to the patient's own home. Care at home is safer and more efficient for some patients, as it decreases the costs and increases patients' life quality. Home care also reduces the usage of the beds and thereby the period of staying at the hospital. Home care applies to individuals of all ages with acute and long-term psychiatric illnesses. In case of changes in the patient's mental status, the essential physical, social and emotional support, preventive, curative, supportive, rehabilitative support, as well as health care and palliative care, are provided. Home care is often an integral component of the post-hospitalization recovery process, especially during the initial weeks after discharge when the patient still requires some level of regular physical assistance [8].

Furthermore, home health care services for psychiatric patients should be initiated one or two months after hospital discharge, and the visits should be once or three times weekly. In most countries, staff who provide home care receive standard training. Specifically, community psychiatric nurses should take at least a one-year master's degree in the field of psychiatry to support patients at home. Nurses recruited for in-home care services must have skills such as mental health assessment, psychological education, cognitive behavioral therapy, symptom management, family or caregiver education, care management, and coordination. Nurses who have these skills will provide more organized and efficient care programs. There are variances between hospital and home care nursing; one of them is the measure of the ability to assess the rate of adverse events occurring after providing the service. The occurrence of adverse effects can be questioned and later given a more systematic study of hospital care and be subject to the audit service. Receiving home care usually takes a more extended time, which affects the rate of adverse effects that may extend to many more years. Blais et al. evaluated the rate of adverse events during home care. The results presented the following statistics: the injuries (17%), wound infections (14%), psychosocial, behavioral, and mental problems (11.8%), and other kinds of adverse events (57.2%) occurring during this time [8]. Functional, neurological, emotional, and behavioral assessment of patients is essential with adequate screening programs to the home care services.

Nevertheless, healthcare services lack suitable screening programs to recognize patients with mental health issues. Consequently, home care services are essential for the quality of life of chronic psychiatric patients and their families. Home care service given to patients according to their determined needs, with adequate evaluation methods, is based on the development of the patient's quality of life by providing them the opportunity to gain their independence. The implementation of such services and care will improve the patient's quality of life and that of the family members [8].

## Materials and methods

This is a retrospective observational study that involves a review of the medical record of patients attending the psychiatry clinics at Shaikh Khalifa Medical City (SKMC) receiving oral second-generation atypical antipsychotics during the study period. The study period is from 1st January 2016 to 31st December 2017. This paper aims to identify the prevalence of metabolic syndrome associated with the use of second-generation atypical antipsychotics in SKMC. Also, it evaluates the prescribing pattern of second-generation atypical antipsychotics (Amisulpride, Aripiprazole, Clozapine, Olanzapine, Paliperidone, Quetiapine, and Risperidone) in adult patients attending psychiatry clinics at SKMC and to evaluate the management of metabolic syndrome associated with the use of second-generation atypical antipsychotics in adult patients attending psychiatry clinics at SKMC.

Inclusion criteria

All adult patients with mental illness who received second-generation atypical antipsychotics from the psychiatry clinics at SKMC during the study period.

Exclusion criteria

Pediatric patients; patients on other antipsychotic, anti-manic, antidepressant, and anticonvulsant medications along with the second-generation antipsychotic; patient with metabolic syndrome history before receiving a second-generation antipsychotic.

Ethical considerations

For this retrospective study, a Protecting Human Research Participants (PHRP) certificate was obtained online through the National Institute of Health website on December 13, 2017, as well as the approval of the Institutional Review Board, Shaikh Khalifa Medical City on 27 February 2018.

Study population

Adult patients with mental illness receiving second-generation atypical antipsychotics from the psychiatry clinics in the Behavioral Science Pavilion, SKMC.

Literature review

For the literature review, many articles were reviewed and obtained from PubMed, American Medical Association, Canadian Journal of Psychiatry, the British Journal of Psychiatry, JAMA Psychiatry, Endocrine Society, Pakistan, Journal of Medical Sciences, Agency for Healthcare Research and Quality, National Heart, Lung, and Blood Institute, and World Health Organization (WHO). In addition to, an extensive review of the British Association for Psychopharmacology (BAP) guidelines on the management of weight gain, metabolic disturbances, and cardiovascular risks associated with psychosis and antipsychotic drug treatment, and the Maudsley Prescribing Guidelines in Psychiatry 12th edition were visited.

Statistical analysis

IBM SPSS v. 25 (IBM Corp., Armonk, NY) and Jamovi 1.0.4.0 (The jamovi project (2021). [Computer Software]. Retrieved from https://www.jamovi.org) were used for statistical analysis. Continuous variables are represented as mean and standard deviation while categorical data are presented as frequencies. Continuous data were tested for normality by the Shapiro-Wilk test. A one-way repeated-measures analysis of variance (ANOVA) was conducted to determine whether there were statistically significant differences between means of BMI at baseline, after six months, and after 12 months of using second-generation antipsychotics. A Kendall's tau-b correlation was run to determine the relationship between the change of BMI at one year and the use of second-generation antipsychotics (single versus combination). Paired t-test was used to determine whether there was a statistically significant mean difference between BMI at baseline and after one year. All statistical analyses were run for patients with no missing values for all the variables used for the test (19 patients for repeated one-way ANOVA, and Kendall’s tau-b, 33 patients for paired t-test). The significance level was set at 0.05.

## Results

Included and excluded patients

A report of patients attending the behavioral sciences pavilion between the 1st of January 2016 and the 31st of December 2017 was obtained. The report revealed that 4123 patients were on second-generation antipsychotics, particularly on: Amisulpride, Aripiprazole, Clozapine, Olanzapine, Paliperidone, Quetiapine, and Risperidone on different available doses. A total of 4000 (97%) patients were excluded due to non-eligibility, as they were either overweight or obese with BMI greater than 24.9 kg/m^2^ before starting the treatment with second-generation antipsychotics, diagnosed with diabetes, dyslipidemia, or another metabolic syndrome, or they were receiving any treatment for previous conditions or pediatric patients. Besides those who received antidepressants, first-generation antipsychotics, mood stabilizers, and other psychiatry medications that are known to cause metabolic disorders were excluded as well.

**Figure 1:**
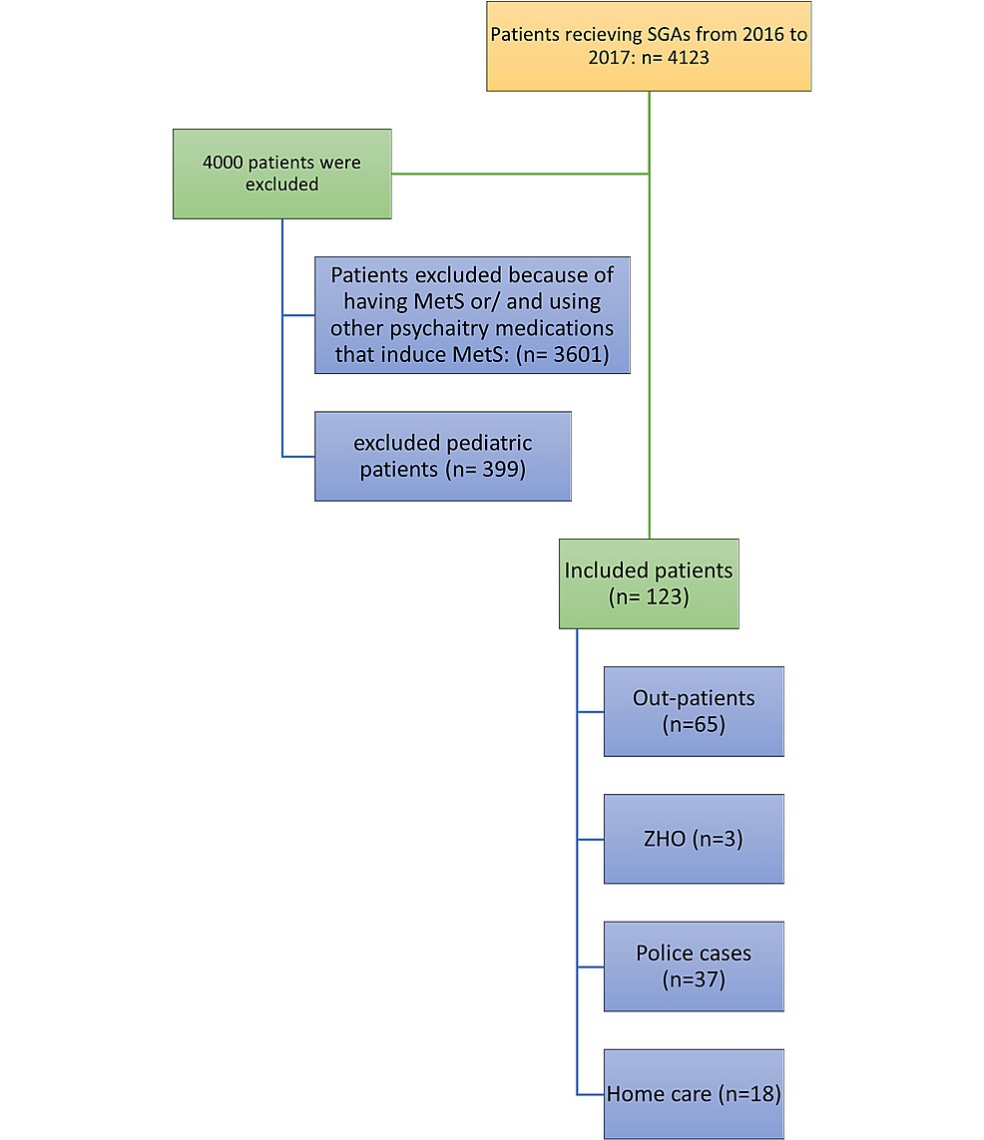
Study population: included versus excluded patients N = number of patients, SGAs = second-generation antipsychotics, MetS = metabolic syndrome, ZHO = Zayed Higher Organization patients

Based on the inclusion criteria, 123 (2.98%) patients were included. Those patients were classified into subgroups including home care patients, patients from Zayed Higher Organization (ZHO), police cases, and regular patients. For the first two subgroups, the nurse is the authoritative person to collect patient medications while for the police cases, the accompanying policeman is responsible to collect the prescribed medications from the pharmacy. Three (2.44%) patients belonged to the ZHO subgroup, 18 (14.64%) patients were under the home care team (CCMT), 37 (30.08%) patients were referred to the behavioral science pavilion as police cases and they received their second-generation antipsychotics from the pharmacy, and 65 (52.85%) patients were regular outpatients (Figure 1).

Demographics and patient diagnosis

**Figure 2:**
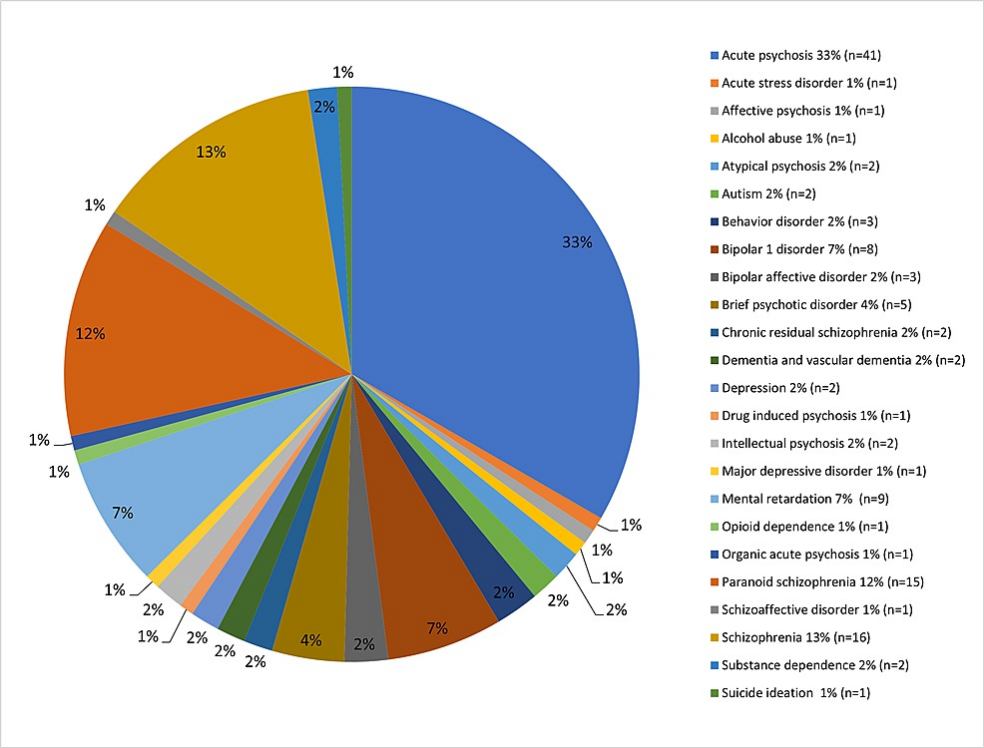
Prevalence of patient’s indications for second-generation antipsychotics use (N=123) N = number of patients

Out of 123 patients, there were 77 (62.60%) male patients and 46 (37.40%) female patients, the male to female ratio was approximately (2:1), and the average age was 35 years old. The youngest patient was 18 years old while the eldest was 86 years old. The minimum duration of treatment was one year compared to the maximum treatment duration of seven years of receiving second-generation antipsychotics from an outpatient pharmacy. Likewise, 24 diagnoses were indicated for the use of such medications, including acute psychosis, acute stress disorder, affective psychosis, alcohol abuse, atypical psychosis, autism, behavior disorder, bipolar 1 disorder, bipolar affective disorder, brief psychotic disorder, chronic residual schizophrenia, dementia, and vascular dementia, depression, drug-induced psychosis, intellectual psychosis, major depressive disorder, mental retardation, opioid dependence, organic acute psychosis, paranoid schizophrenia, schizoaffective disorder, schizophrenia, substance dependence, and suicide ideation (Figure 2).

Patient treatment regimens

**Figure 3:**
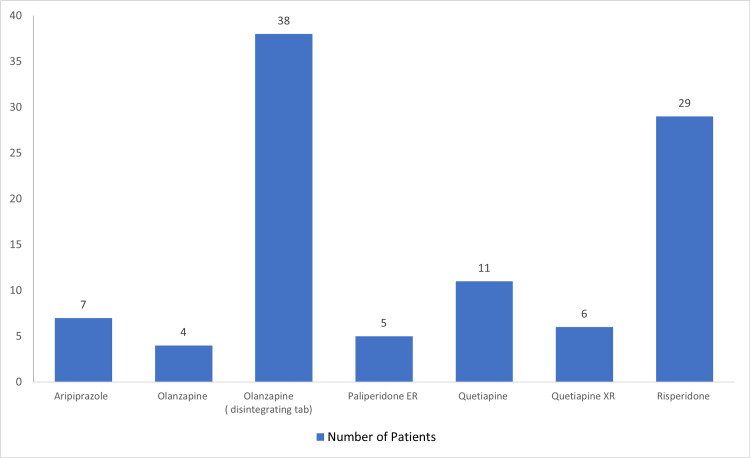
Prevalence of patients on a single agent of second-generations antipsychotics (N=100) ER/XR = extended release

**Figure 4:**
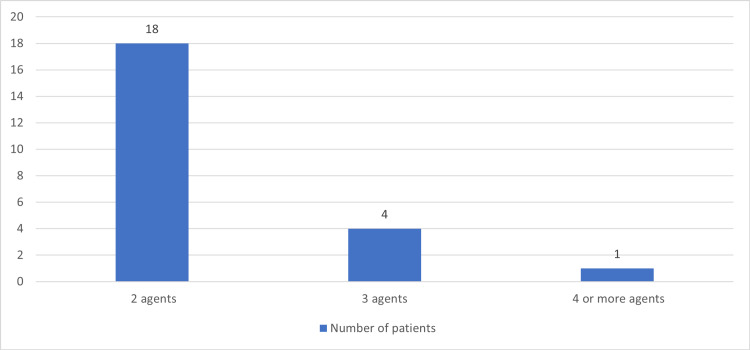
Number of patients using more than one agent of second-generation antipsychotics (N=23)

A total of 100 (81.30%) patients were on a single second-generation antipsychotic regimen as compared to 23 (18.70%) patients who were on combinations of second-generation antipsychotics (Figures 3-4). Aripiprazole appeared in 11 (8.94%) patients’ regimens in combination with other atypical antipsychotics while olanzapine and olanzapine disintegrating tablets appeared in 14 (11.38%) regimens along with other atypical antipsychotics. On the other hand, Quetiapine (extended-release) tablet - Quetiapine tablet appeared in 10 (8.13%), paliperidone ER in seven (5.69%), risperidone in six (4.88%), and finally, amisulpride was combined with other second-generation antipsychotics only in one (0.81%) patient regimen.

Prevalence and management of metabolic disorders

**Figure 5:**
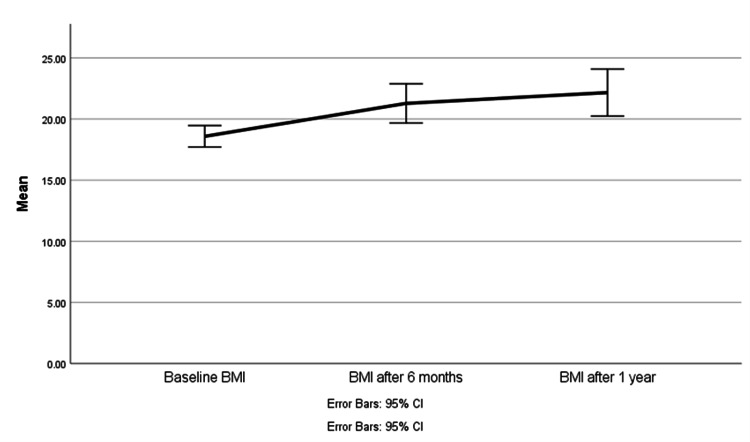
Change in mean BMI at baseline, six months, and 12 months

The correlation between second-generation antipsychotics and different variables, including BMI, HbA1c, total cholesterol (TC)/high-density lipoprotein (HDL), and blood pressure at baseline, six months, one year, and last documented measures were assessed. At the baseline, the BMI of 20 (16.26%) patients were less than 18 kg/m^2^, the documented BMI for 79 (64.23%) patients were between 18 and 24.9 kg/m^2^ and it was found that 24 (19.51%) patients have no documented BMI, weight, or height to estimate the BMI. While an after one-year BMI of less than 18 kg/m^2^ was documented for three (2.44%) patients, 21 (17.07%) patients' BMI was between (18-24.9 kg/m^2^), and 91 (73.98%) patients had incomplete data with regards to the BMI. The Shapiro-Wilk test has shown that all continuous variables tested were normally distributed ( p>0.05). The weak correlation between the difference of BMI after one year and the use of single or combination was not statistically significant (τb = .022, p = .911). A one-way repeated-measures analysis of variance (ANOVA) was conducted to determine whether there were statistically significant differences in BMI over the course of a 12-month use of SGA. There were no outliers, and the data were normally distributed, as assessed by the boxplot and Shapiro-Wilk test (p > .05), respectively. The assumption of sphericity was not violated, as assessed by Mauchly's test of sphericity, W = 0.716, p = .059. The use of SGA resulted in increased BMI, F (14.5) = 26.938, p < .001, partial η2 = 0.446, with BMI increasing from baseline (M = 18.9, SD = 5.8 kg/m^2^) to six months (M = 21.3, SD = 3.3 kg/m^2^) to 12 months (M = 22.2, SD = 4 kg/m^2^). Post-hoc analysis (Tuckey) with a Bonferroni adjustment revealed that BMI was statistically significantly increased from baseline to six months (M = 2.37 kg/m^2^, p = .002) and from baseline to 12 months (M =3.26 kg/m^2^, p < .001) but not from six months to 12 months (M = 1.41 kg/m^2^, p = .346) (Figure 5). The patients’ BMI at baseline (M = 19 kg/m^2^, SD = 2.28) was less than BMI after 12 months of using SGA (M = 22 kg/m^2^, SD = 3.87), a statistically significant mean increase of 3 kg/m^2^, 95% CI [-4, -1], t= -4.56, p < .001. The current BMI of average three years of receiving second-generation antipsychotics was as follows: three (2.44%) patients with less than 18 kg/m^2^, 21 (17.07%) with BMI fall between 18 and 24.9 kg/m^2^, and 35 (28.46%) patients with BMI above 24.9 kg/m^2^. Likewise, the average observed increase between BMI readings from baseline after six months of receiving the treatment and one-year BMI: 1.15 kg/m^2^ and 1.75 kg/m^2^, respectively. Hence, the clinical significance cannot be estimated due to a lack of data. Similarly, documented variables at six months duration (HbA1C, TC/HDL) after receiving the therapy were not tested, as they cannot be statically computed according to the missing data. The systolic blood pressure (SBP) of patients was 121.5 mmHg (± 10.7) while diastolic blood pressure (DBP) was 74 mmHg (± 7.5).

Finally, nine (7.32%) patients were known to have metabolic syndrome after being on second-generation antipsychotics, and treatment was initiated. Treatment included anti-diabetic agents and blood-glucose-lowering agents (metformin, insulin, and gliclazide), lipid-lowering agent (atorvastatin), and anti-hypertensive medications (propranolol and valsartan). Metformin was added for five patients, gliclazide for two patients, and insulin for one patient. While propranolol was added for three patients and valsartan for two patients, atorvastatin was prescribed for two patients only to treat dyslipidemia.

## Discussion

Obesity, overweight, and risk of cardiovascular diseases (CVD)

Obesity has been recognized as a global epidemic in both adults and adolescents and is associated with various comorbidities, including hypertension, type II diabetes mellitus, dyslipidemia, obstructive sleep apnea, certain cancers, and major cardiovascular (CV) diseases. Because of its maladaptive effects on various CV risk factors and its adverse effects on CV structure and function, obesity has a significant impact on CV diseases, such as heart failure (HF), coronary heart disease (CHD), sudden cardiac death, and atrial fibrillation, and is associated with reduced overall survival [19]. Furthermore, several studies have established the connection between high BMI and CVD risk in several populations. The association between high BMI and overall risk for developing coronary heart disease (CHD) has increased by 20% in overweight individuals and 50% in obese individuals; both overweight and obesity are associated with increased risk for developing atrial fibrillation and stroke. In addition to that, an increase of 1 kg/m^2^ in BMI results in an 8.4% increase in the risk of developing diabetes [19].

Antipsychotic medications and weight gain management

Overweight and obesity, partly driven by antipsychotic drug treatment, are essential factors contributing to the development of diabetes and cardiovascular disease in people with schizophrenia. Additionally, deaths from cardiovascular disease are a chief contributor to the significant lessening in life expectancy experienced by people with schizophrenia. Likewise, there have been clinical trials of many interventions for people experiencing weight gain when taking antipsychotic medications but there is a lack of clear consensus regarding which may be appropriate in usual clinical practice.

Hence, the monitoring of relevant physical health risk factors is frequently inadequate. The British Association for Psychopharmacology guidelines on the management of weight gain, metabolic disturbances, and cardiovascular risks linked with psychosis and antipsychotic drug treatment, recommended in their guidelines some measurements that should be assessed before starting an antipsychotic, or as soon as possible afterward, and then at the intervals indicated [20].

BMI should be used to monitor whether an individual is becoming overweight or obese, which requires frequent measurement of weight during the early stages of treatment: ideally weekly for the first four to six weeks and then every two to four weeks up to 12 weeks, but, as a minimum, once every four weeks for the first 12 weeks of treatment. Weight and BMI should then be assessed at six months and at least annually after that unless the clinical situation requires a more frequent assessment. Moreover, if there is a change in antipsychotic medication, then, when clinically relevant, it is appropriate to revisit all the previous steps. Keep in mind that it is crucial to take ethnicity into account when evaluating BMI results [20]. An audit performed by POMH revealed that 25% of those with elevated BMI developed high blood pressure. BAP guidelines recommend employing such variables to monitor and predict the risk of antipsychotics in the general population. Moreover, blood pressure should be monitored at 12 weeks, six months, and annually after that. In case of hypertension development, it should be managed according to standard National Institute for Health and Care Excellence (NICE) guidelines and practitioners should be aware of possible increased hypotensive effects when some antihypertensive medications are combined with antipsychotics [20].

So, for those who developed obesity and an increase in weight after using second-generation antipsychotics, there are many recommendations and interventions to manage medication-induced obesity [20]. Lifestyle interventions are recommended, and they should always be the first-line approach in most circumstances and should be continued in addition to any additional intervention. Antipsychotic switching is another intervention to be considered. Clinicians must balance the possible benefit on the weight of switching antipsychotic medication against the risks of inducing relapse of psychotic symptoms. Many meta-analyses of the differential effects of different medications on weight support antipsychotic-switching strategies. These data suggest a hierarchy of antipsychotic medications concerning weight gain, with the following medications appearing to carry the lowest tendency for weight gain: haloperidol, ziprasidone, lurasidone, aripiprazole, amisulpride, and asenapine [20].

Furthermore, another intervention is using aripiprazole as adjunctive therapy with certain atypical antipsychotics. Adjunctive aripiprazole is recommended as a possible intervention for weight gain associated with clozapine and olanzapine. Three randomized control trials of the addition of aripiprazole to clozapine or olanzapine were published, only one of significant size, found a mean difference in weight loss for aripiprazole over placebo of just over 2 kg. What is more, in the context of recommendations regarding groups at high risk of diabetes inNICE) Public Health 38 guidance, metformin can be used as an adjunct as well for weight reduction following antipsychotic medication [20].

Also, glucagon-like peptide-1 (GLP-1) receptor agonists are useful for weight reduction in obesity in the general population, and liraglutide has been given marketing authorization for this use [21]. However, there are no randomized control trial data yet available for its use in people with psychosis taking antipsychotic medications. Orlistat has been subject to randomized control trials in the general population, where it reduces weight by approximately 3 kg over one year. Though high rates of discontinuation limit long-term use [20].

Furthermore, three out of four randomized control trials of using topiramate as an adjunct to antipsychotics reported statistically significant weight loss, ranging from 1.5 kg to 5 kg. One randomized control trial supports an effect to lessen weight gain in people with the first episode of psychosis. However, the risk-benefit profile of topiramate is severely limited by its adverse effects. On the other hand, reboxetine use for weight management with atypical antipsychotics has consistent data from three trials suggesting benefit, but all are from a single research group with no independent replication yet available. Compared to amantadine, melatonin, and zonisamide use for the same indication, they have all been subject to randomized control trials that suggest a beneficial effect. However, available data are too limited to make any recommendation regarding their use. Finally, clinical trials of atomoxetine, dextroamphetamine, famotidine, fluoxetine, fluvoxamine, and nizatidine have failed to show benefit in reducing weight induced by atypical antipsychotics [20].

An audit performed by Prescribing Observatory for Mental Health (POMH) revealed that 25% of those with elevated BMI developed high blood pressure. BAP guidelines recommend employing such variables to monitor and predict the risk of antipsychotics in the general population. Moreover, blood pressure should be monitored at twelve weeks, six months, and annually thereafter [20].

Second-generation antipsychotics and diabetes management

A feature of metabolic pathology of psychosis is increasing the risk of diabetes by two-folds in psychotic patients. Such an increase is still complex, and it is associated with genetics, lifestyle, and the use of atypical antipsychotics. The risk of diabetes in a patient with schizophrenia was compared to the general population in an observational study, and it showed that the risk of diabetes is higher among people receiving antipsychotic medication and is higher in people with schizophrenia who have experienced multiple episodes as compared to first-episode and untreated individuals. It was noticed that antipsychotics are likely increasing the risk of diabetes through weight gain, as this will subsequently increase insulin resistance [20].

As per BAP guideline recommendations, in the long term, blood glucose control should be monitored using glycated hemoglobin (HbA1c) [20]. However, as HbA1c provides a measure of longer-term control, in the early weeks of treatment, fasting or random plasma glucose may offer a more appropriate measure of glucose control. Moreover, glucose control should be further assessed at 12 weeks, six months, and then annually. The management of the medical consequences of weight gain and obesity is the first step, and it should be in the primary care service. Hence, initial investigations may be done either by the mental health team or the primary care team. For those with potential prediabetic states, the patient should be investigated and managed as per NICE guidelines. Lifestyle modification is the primary intervention. For those who are not responding to intensive lifestyle interventions, a prescription of metformin may be considered. Though there are some risks attached to metformin use that require appropriate monitoring such as renal function and vitamin B12 [20].

Monitoring of lipid profile and management dyslipidemia

Lipid profile should be assessed at 12 weeks, six months, and then annually. In order to assess cardiovascular risk, the TC/HDL cholesterol ratio assessment is required. A random sample rather than a fasting sample can be used if a fasting sample cannot be obtained. Besides, the NICE clinical guidelines on schizophrenia and bipolar disorder recommend lipid levels monitoring, including cholesterol in all patients aged 40 years or over, even if there is no other indication of risk. Management will usually be as per current guidelines such as the NICE guidelines on lipid modification or the JBS2 Joint British Societies' guidelines on the prevention of cardiovascular disease in clinical practice that recommends the following: all people with dyslipidemia should receive advice about diet, exercise, weight management, and smoking cessation. Those with a total cholesterol concentration of over 9.0 mmol/l or a non-HDL cholesterol concentration of more than 7.5 mmol/l or triglyceride concentration of more than 10 mmol/l should be referred to specialist advice. Regarding statin prescribing, NICE recommended that if lifestyle advice is ineffective in normalizing the lipid profile, a statin should be considered after screening for their risk of CVD since there is no contraindication of the prescription of a statin in people prescribed antipsychotics. Primarily atorvastatin should be offered for the primary prevention of CVD to people who have a 10% or greater 10-year risk of developing CVD; generally, people in these categories will be over 40 years of age. Statins should also be offered for the primary prevention of CVD to people with type 2 diabetes who have a 10% or greater 10-year risk of developing CVD as well. Furthermore, dyslipidemia, especially in the context of a person with diabetes, should be actively managed according to existing NICE guidelines for the general population. Besides previous interventions, switching to other second-generation antipsychotics with a lower liability of causing dyslipidemia could be considered as an intervention. Focusing on the lipid-lowering aspect, four studies identified by a Cochrane Systemic Review concluded that switching to aripiprazole resulted in an improved lipid profile [20].

Second-generation antipsychotics and prolonged QT interval

Drug-induced QT prolongation is associated with a higher risk of arrhythmia and cardiovascular mortality. The risk of QT prolongation is associated mainly with first-generation antipsychotics rather than the second-generation. It was documented that some second-generation antipsychotics induce QT prolongation and result in greater odds of Torsades de pointes. In addition, high dose anti-psychotics treatment (HDAT) in case of emergencies plus a combination of antipsychotics increase the risk of QT interval prolongation. Subsequently, ECG is recommended as the only pretreatment when needed, at an increased dose of first-generation antipsychotics, in combination regimens with one or more antipsychotics, and when other medications known to induce QT prolongation are used along with antipsychotics. In such cases, ECG should be performed in order to exclude cardiac contraindications. When ECG is not performed, the reason must be clearly documented in the patient medical profile. Cardiac contraindications such as a history of CVD, family history of CVD, clinical examination showing an irregular pulse, and if the patient has factors that may predispose arrhythmia-like systemic disease or electrolyte abnormalities besides potential drug-drug interaction that might increase the plasma level of antipsychotics or drugs that might prolong the QT interval, causing arrhythmia, should be checked before HDAT initiation [22-23]. Focusing on second-generation antipsychotics, low-quality evidence suggests that aripiprazole and olanzapine are not inducing QT interval prolongation while ziprasidone increases the QT interval moderately and both quetiapine and risperidone are associated with a greater risk of QT prolongation and Torsades de pointes events [24].

Community case management team (CCMT) and homecare patients

CCMT is a group of multidisciplinary team members who provide psychiatric community care to the most vulnerable population suffering from serious mental illness in their environment. CCMT provides comprehensive psychiatric care to patients at their home within a culturally relevant context, promotes mental health through community education designed to increase public awareness of mental illnesses and to decrease the psychiatric stigma, help patients live independently, and reduce relapse frequency, in addition to crisis intervention in collaboration with the authorities. The team of CCMT consists of at least two members: a psychologist, a social worker, or a psychiatrist nurse headed by a psychiatrist. They provide a variety of services: follow-up by a psychiatrist, supportive psychotherapy, family therapy, counseling, health education, and long-acting depot injections. There are specific policies for a CCMT service, including claim management, documentation, infection control, medical equipment management, medical waste management, and medication management, as they follow Abu Dhabi Health Services (SEHA) and SKMC medication policies and blood collection policy [25]. These services include training and education, vocational and therapeutic rehabilitation, psychological care, family counseling, as well as support and education.

Importance of clinical documentation and monitoring

Focusing on patient data and monitored parameters documentation, clinical notes should be well thought out, be written, and give a detailed picture of the clinical situation. They must include relevant information, including the previous measurements, the patient’s history, the source of information, and the date and time of the encounter. Additionally, relevant history with pertinent positive and negative findings should be clearly stated in the medical records [26].

Through the data collection phase and data analysis, it was noticed that there are a lot of missing data in regards to measurements and monitoring of second-generation antipsychotic’s impact on the lipid profile, the glucose level in the short and long term, and even weight and BMI. What is more, it was clearly observed that TC/HDL was measured only for seven (5.69%) patients at the baseline and for three patients after one year of receiving second-generation antipsychotics while it was not checked for any patients after six months of the treatment. Moving to the blood glucose level, the random glucose level was checked only for 51 (41.46%) patients out of 123 at the baseline while it was not measured for any patients after receiving the treatment for six months, and the level of HbA1c was measured for two (1.63%) patients after one year of treatment. Such findings reflect on either poor documentation or lack of monitoring, which can lead to severe consequences. Since the policy of homecare supports treatment monitoring and patient education, it is highly advised to reinforce the role of treatment monitoring and follow up with home care patients regularly. In addition, nurses from ZHO are responsible for medication collection from the pharmacy, and it is believed that nurses are playing a vital role in medication administration and treatment monitoring for this population [27].

## Conclusions

In conclusion, it was noticed that an increase in one-year measured BMI was highly associated with second-generation antipsychotic use. Moreover, there is a proportional relationship between the use of second-generation antipsychotics and BMI rises. It is known that increased BMI leads to a significant increase in cardiovascular disease, atrial fibrillation, stroke, and diabetes risk. Likewise, interventions and treatment monitoring are essential in managing those patients. Individualized management to prevent further complications is highly advised. Furthermore, documentation and treatment monitoring are cornerstones in the patients’ treatment.

This study reveals a gap in treatment monitoring and documentation, which affects testing research variables. It is highly recommended to initiate an outpatient internal medicine clinic as an outpatient service in the Behavioral Science Pavilion, as it will enhance the patient's outcome and will enhance the level of monitoring. What is more, reinforcement and re-education of homecare policy is a must to ensure care continuity in addition to the development of clear guidelines and monitoring policies with such agents. It is highly recommended to do further research and studies in order to maximize the provided level of care and minimize the complications.
